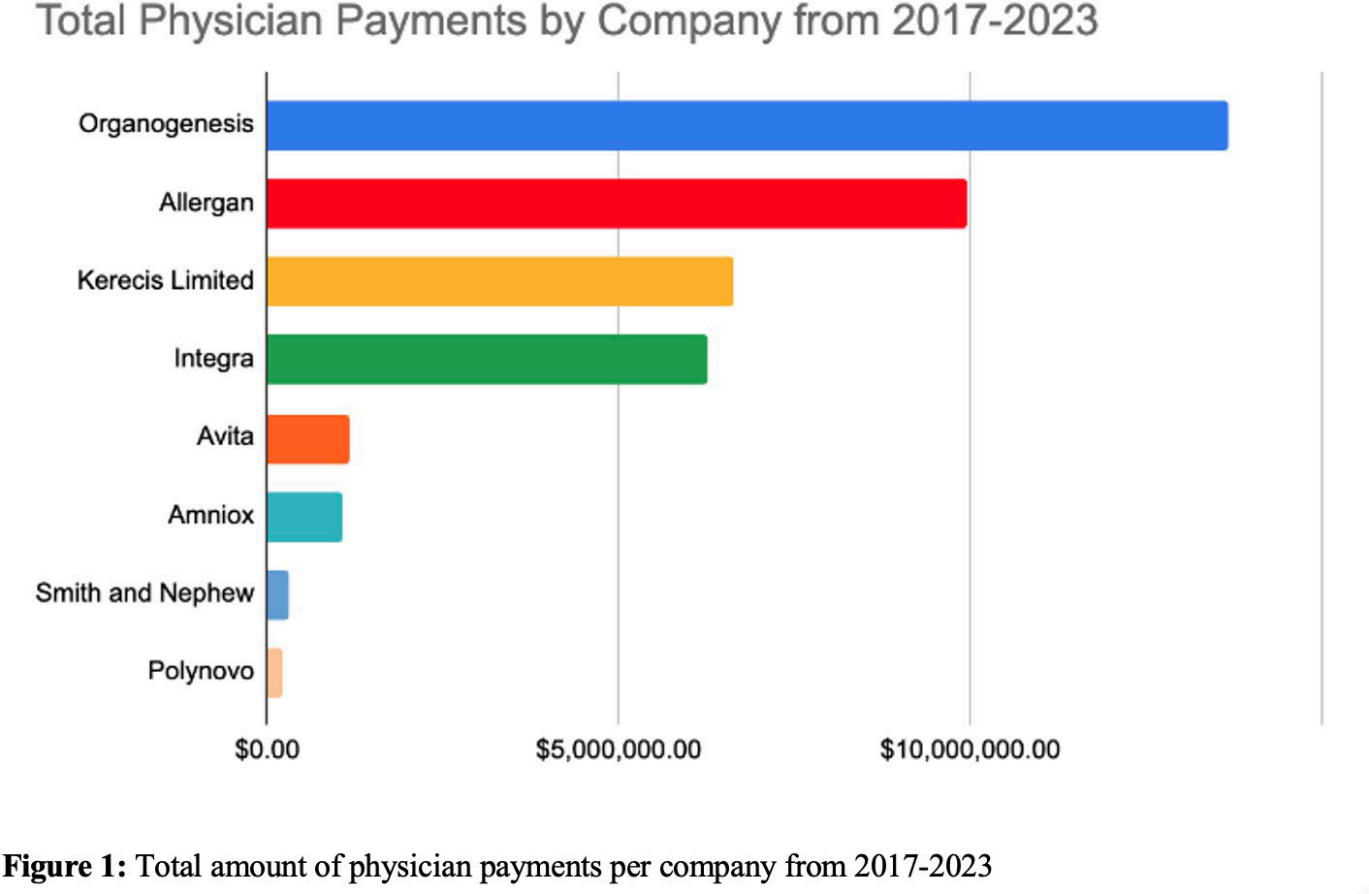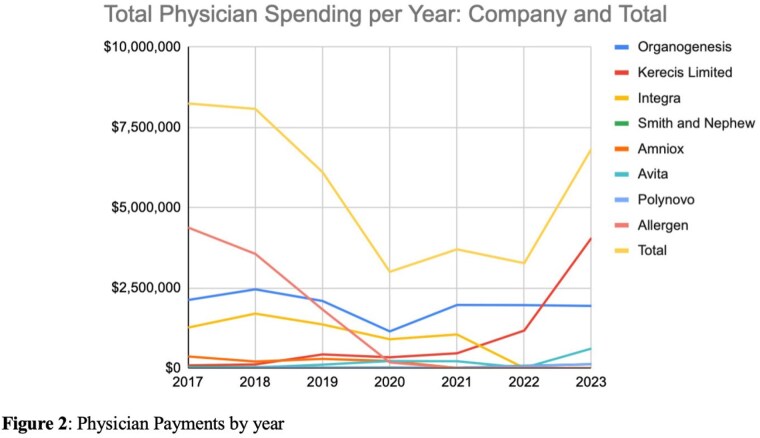# 17 Physician-industry Payments in the Skin Substitute Market—Shedding Light from the Sunshine Act

**DOI:** 10.1093/jbcr/iraf019.017

**Published:** 2025-04-01

**Authors:** Chandler Hinson, Mollie Smith, Clifford Sheckter

**Affiliations:** University of Texas Southwestern; University of Massachusetts Chan Medical School; Santa Clara Valley Medical Center

## Abstract

**Introduction:**

Skin substitutes are important in caring for burn patients, and industry provides a vital partner in innovating and supplying these products. Burn physicians are often engaged in discussing and promoting these products. The Physician Payments Sunshine Act mandates transparency in reporting industry payments to healthcare providers. These payments, which include consulting fees, speaking engagements, and research support, have the potential to influence clinical decision-making and product selection. This study aims to analyze current trends in physician-company financial relationships within the skin substitute market, providing insights into the extent and nature of these interactions.

**Methods:**

Companies with the largest market share of skin substitutes and burn wound coverage products (ex. autologous cell harvesting) were selected. Each company’s public payment records to physicians were extracted from OpenPaymentsData.CMS.gov from 2017-2023. Total yearly payments per category were analyzed. Types of payments included: compensation for serving as faculty or as a speaker for a non-accredited and non-certified continuing education program, consulting fees, education, food and beverage, and travel and lodging.

**Results:**

Eight companies encompassing 26 products were included in this study (Figure 1). There was variable spending per year including a notable decline during the COVID-19 pandemic (2020-2021) (Figure 2). Excluding the pandemic, total annual payments paid to physicians ranged from $0 to $4,379,2420.77 with a median of $115,052.45 and an interquartile range (IQR) of $8,040.10 to $1,442,151.53. Per physician per year, payments ranged from $0.35 to $1,088,405.68 with a median $47.17 and IQR of $21.44 to $130.57. By category, the greatest spending on physicians across companies was for “compensation for serving as faculty or as a speaker for a non-accredited and non-certified continuing education program” and “consulting fee,” with an average of $2,741,643.65 and $1,046,408.71 spent per year, respectively. Spending was not consistent across companies, with only two companies (Kerecis and Avita) having the only significant increase in direct physician payments in the past 3 years.

**Conclusions:**

Payments to physicians varied widely with some physicians earning upwards of $1,088,405 per year. While these numbers are not disclosed during promotional events for products, these payments may influence a speaker’s promotion of a particular product or brand, raising concerns about whether an endorsement is based on genuine clinical efficacy or financial self-interest.

**Applicability of Research to Practice:**

Industry is an important partner in innovating and supplying creative solutions for burns. Financial conflicts need to be highlighted in the physician promotion of products and the research from these physicians. The ABA could consider detailed financial disclosures during presentations from these industry sponsored physicians.

**Funding for the Study:**

N/A